# Political parties matter: the impact of the populist radical right on health

**DOI:** 10.1093/eurpub/cky157

**Published:** 2018-11-01

**Authors:** Michelle Falkenbach, Scott L Greer

**Affiliations:** 1 PhD Scholar, University of Michigan, Ann Arbor, Michigan, USA; 2 Professor of Health Management and Policy, Global Public Health and Political Science, University of Michigan, USA

## Abstract

This paper presents the basic political science consensus on parties and their impact on policy, then turns to focus on the impact of the populist radical right (PRR) parties on policy, what PRR parties have done to implement their views and whether they make a difference. Three effects on policy were established: 1) they de-emphasize the issue, preferring to focus on migration, crime and security rather than health and welfare and 2) they prefer to pursue exclusionary policies. 3) it is not clear whether they increase or decrease benefits for the “native” populations they claim to represent. In short PRR parties make a difference whether to migrants or conservative governments, this party group matters.

Political parties are at the center of politics. They coordinate politicians, mobilize voters, shape identities and collaborate across borders. However unloved parties and party systems might sometimes be, modern democratic politics is unthinkable without them. But, perhaps surprisingly to many, there are real debates about their impact on policy. Do parties make a difference? This article presents the basic political science consensus on parties and their impact on policy, then turns to focus on the impact of the populist radical right parties on policy. Its objective is to show the importance of political parties and some of the factors that shape their impact, in the particularly timely case of the PRR parties.^1^

A *political party* is ‘any political group that presents at elections, and is capable of placing through elections, candidates in public office’.^2^ Parties serve crucial functions.^3^^,^^4^ They coordinate politicians within legislatures and between different (e.g. local and central) governments, structure political careers and recruitment, create networks of diffuse reciprocity between politicians over time and provide labels voters can understand. Collectively, parties form *party systems*. A party system is simply the sum of parties and their relationships to each other, typically mapped in some sort of ideological space (e.g. left-right). Parties in Europe, and the world, form families based on their shared predispositions, such as social democratic, liberal, Christian Democratic or left (e.g. Syriza, Podemos, Die Linke), and often coordinate their actions across borders.

## Do parties make a difference?

This is a classic question of political science and one with obvious implications for anybody interested in policy and social change.^5^ Scholars asking this question address health issues through a broad approach to social policy and comparative politics, which sees generous and egalitarian welfare states, including health systems, as an effect of strong left parties linked to strong trades unions.^6^ This literature typically finds that vote shares and control of government for left parties lead to more generous and egalitarian welfare states, while Christian Democratic parties have supported large but inegalitarian welfare states.

There are problems with this conclusion, however. It can be critiqued for the use of time-series analysis on data with enormous fixed country effects. Its record when tested with detailed qualitative studies is also poor, and shows just how complex the relationship can be. Sara Watson, for example, found that the presence of a strong left party (as with communists in Portugal and Italy, or Die Linke in Germany today) leads to weaker social protection because center-left parties refuse to collaborate with the left party and instead negotiate with the center-right.^7^ A strong and solid left-wing party vote, in these important but counterintuitive cases, can push social policy to the right.

As a result, there is a paucity of detailed country-level evidence for the overall quantitative finding, which is troubling and suggests a need for further research. Too many country experiences that diverge from the statistical results means we should question the statistical results. And if we have yet to work out the impact of social democratic parties on overall social policy, there is much work to do if we are to understand the impact of, for example, populist right parties.

## The significance of populist radical right parties

Populist radical right (PRR) parties have been gaining votes and prominence across Europe in recent years, causing much concern about their likely impact on health and health policy. PRR parties are nativist (believing that there is an ethnically united people with a territory, aka nationalism or ethnocentrism), authoritarian (believing in the value of obeying and valuing authority) and populist (preferring the ‘common sense’ of a unified people to elite knowledge).^8^ They include, among others, the French National Front, the Austrian Freedom Party (FPÖ), the Italian Northern League, the Alternative for Germany (AfD), the Polish Law and Justice (PiS) party, the Dutch Party for Freedom (PVV) and the Sweden Democrats.

**Figure 1:**
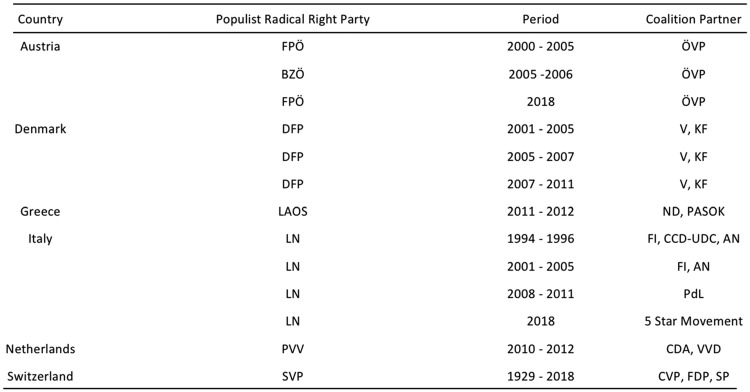
PRR parties in government—updated from Mudde (2013)

Since the 1960s, the PRR vote in both national and European parliamentary elections has more than doubled (5.1–13.2%) at the expense of center parties and their share of seats in parliament have tripled (3.8–12.8%).^9^ This increase in votes has not yet been matched by participation in government. More than 200 national governments in Western Europe have been formed since 1980, but only 13 of them have included PRR parties, almost all as junior partners (figure 1). Although PRR parties have not been in government in most countries, the frequency of PRR government participation is increasing and public health experts need to be aware of what this means.

Particularly in Western Europe, the rise of PRR parties ‘is a reaction to the failure of traditional parties’ ability to respond adequately in the eyes of the electorate’ to issues surrounding mass migration and financial insecurity.^10^ PRR parties often claim to protect citizens’ social welfare benefits, including healthcare, from non-citizens (mostly migrants). Rhetorically, most endorse a model known as *welfare chauvinism*, which emphasizes generous and often increased benefits for ‘the people’ and reduced benefits for outsiders.^11^ In recent years, PRR parties have created a false narrative that criticizes mainstream parties for cutting welfare in order to deliver benefits to immigrants.^12^

While the rhetoric of PRR parties is often welfare-chauvinist, they will frequently blur their positions in order to appeal to various different categories of voters.^13^ In practice, it is not clear that they actually pursue welfare chauvinism, as against *liberal chauvinism* that combines exclusion of outsiders from benefits with cuts to benefits for the insiders.^14^ The success of PRR parties has been attributed by some researchers to precisely this combination of nationalism and neoliberalism.^15^

There is an urgent need for research that will allow us to understand when and how PRR parties incline toward welfare chauvinism or exclusionary liberalism, for that will determine much of their effect on policies, agendas, health systems and health. In Austria, for example, the impact of the PRR on politics since 2017 has mostly been liberal chauvinist rather than welfare chauvinist—seeking a balanced budget with cuts across multiple programs, though especially ones perceived as serving immigrants, and a rollback of tobacco control legislation (Box 1).

### When do PRR parties shape policy?

**Figure 2:**
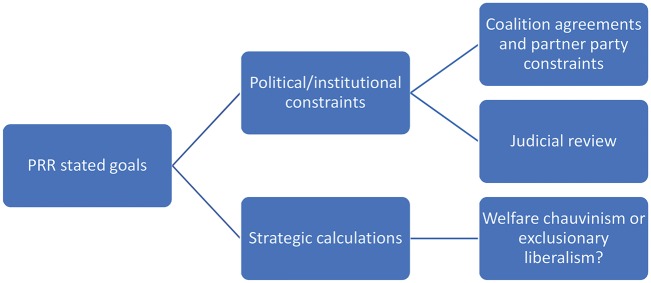
Impact of the PRR on policy (source: author)

There are many factors that influence the impact of a political party on policy. Figure 2 summarizes the key factors standing between PRR parties, in particular, and policy impact. On one side, there is the set of *political and institutional constraints* on the PRR party. Electoral rules determine the effective number of parties in a party system, with proportional representation systems increasing the number of parties. The effective number of parties in most party systems has been going up in most European countries for some time, regardless of electoral rules, as party systems fragment and the big central social democratic and Christian democrat parties decline. In party systems with more parties, governments require coalitions. The PRR parties in European national governments have almost all entered government in coalition with established conservative, mostly Christian Democratic, parties (the exception is Italy). There are very few examples of the social democrats in any country working with the PRR. In coalition government, both the coalition agreement and the partner party constrain what the PRR party can do to pursue its goals—for example, welfare chauvinist objectives might be turned into exclusionary liberalism if the conservative coalition partner, for example, is willing to endorse anti-immigrant policies in return for PRR support for budget cuts. This might be what has happened in Austria. This factor has a further implication. So far the PRR has only entered government in coalition governments in countries with proportional representation. In countries whose institutions make them more prone to have single-party governments with extensive power, such as the United Kingdom or France, a PRR government could be unconstrained and very powerful.

The other major external constraint is the *rule of law*, especially constitutional judicial review but also lower forms of law such as administrative public law review and international law such as that of the European Union. PRR policies, whose explicit goal is often discrimination, can run afoul of rights protection and antidiscrimination law. The effectiveness of courts and the strength of rights protection varies widely and courts rarely can face down an elected government bent on undermining them, but in the short run they can block PRR policy initiatives.

The third factor affecting the impact of the PRR is simply their actual, revealed, *policy preferences*. It is harder to blur goals in government than in campaigning. In the example above, PRR parties might decide that anti-immigrant policies are more important to them and their voters than generous welfare states. Even in an age of weak party organizations, PRR parties are often particularly top-down and short on activists (the name and logo of the Five Star Movement in Italy are literally the property of its leaders Beppe Grillo and Gianroberto Casaleggio while the Dutch PVV has only two members: its leader Geert Wilders and an association he controls) so they have considerable latitude to make even risky decisions.

These three factors are among the many that shape the impact of parties on policy in general, but they have been the dominant ones in the cases of the PRR that we have so far. The PRR is of course also shaped by a force that shapes all parties, which is policy legacies. For example, it is hard to impose exclusionary new laws in self-governing social insurance systems such as that of Austria. All other things being equal, we should expect that the formal impact of the PRR on access to healthcare for legal immigrants should be greater in NHS systems where the eligibility rules and administration of social insurance do not form obstacles.

What have PRR parties done to implement their views while in power? Given the small number of PRR parties in national governments and the complexity of their effects, it is customary to use comparative qualitative analysis to study them.

In Austria, many of the proposed initiatives by the FPÖ in previous governments (2000–2006), such as immediate expulsion of foreigners, were deemed unconstitutional, never translated into policies or blocked by their coalition partner, the Christian Democratic ÖVP.^16^ Healthcare was not featured in the PRR party’s agenda but the party has continued to promote a welfare chauvinist agenda across other policy areas while also supporting cuts promoted by its coalition partners (Box 1).^17^

**Box 1 The PRR in Austria**

Since 2017, Austria has been governed by the PRR (FPÖ) and conservative parties (ÖVP) acting in coalition. The coalition’s goal is to make spending cuts to the welfare system, specifically in relation to migrants, in order to achieve a ‘zero deficit’. The impact of this strategy on health and social policy has been significant. The budget for the unemployment service (AMS) has been reduced from €1.94 billion Euros for 2018 to €1.41 billion for 2019, ending two employment programs (‘Aktion 2000’ and the ‘Beschäftigungsbonus’) supporting the long-term unemployed and elderly in the workforce, and cutting the money spent on integration services by €105 million (ORF, 2018a). Plans to reduce the guaranteed minimum income and cuts to hospital and research budgets have also been announced. But perhaps the clearest impact of the FPÖ is in tobacco control. One of the conditions that the FPÖ presented during coalition talks with the ÖVP in late December 2017 was to drop the ban on smoking due to be implemented in May 2018, which would have prohibited smoking inside restaurants and bars across the country. The ÖVP supported this ban under the social democratic party (SPÖ) government and several prominent members of the party publicly opposed the decision to renege on the agreement, but to no effect.

*Sources:* ORF. AMS-Verwaltungsrat Beschließt Förderbudget 2018 – Kürzung Niedriger. Austria: der Standard, 2018a. https://derstandard.at/2000076888610/AMS-Verwaltungsrat-beschliesst-Foerderbudget-2018-Kuerzung-niedriger.

ORF. Ordensspitäler Bekommen Weniger Geld” *ORF Wien*, 2018b. http://wien.orf.at/news/stories/2899394/.

When we look at Italy, we see a liberal exclusionary impact of the PRR on health and welfare. Many of the initiatives proposed by the Lega Nord (LN), Alleanza Nationale (AN) and the Popolo della Liberta (PdL) contravened the European Human Rights Convention and rulings of the European Court of Justice.^18^ What the 2001 coalition was able to enforce was its exclusionary approach to the distribution of social services by limiting access to services for those not deemed to have contributed enough. At the time of writing (August 2018) the PRR parties in the newly formed Italian government have made their exclusionary intentions clear but it is less certain whether they will produce any expanded health benefits for citizens.

In Switzerland, the PRR party (SVP) has been in office for a long time. It regards health and welfare policies to be unsafe issues and prefers instead to focus on immigration and public safety.^13^ While the SVP chose to de-emphasize broader social policy, it tended to oppose further expansions of the welfare system and favored limited government involvement and assistance.^19^

The Danish PRR party (DFP) very clearly influenced social policy between 2001 and 2011 by steering the country in a welfare chauvinist direction.^20^ Measures restricting access to benefits or lowering the level of benefits for immigrants were introduced, a ceiling on social benefits was implemented, social assistance benefits for those under 25 were reduced and access to social assistance benefits were made conditional on an integration examination. The Danish PRR party has pushed remarkably authoritarian, nativist ideas onto the agenda, from confiscating refugees’ valuables to seizing children from designated immigrant ‘ghetto’ areas.^21^

Initially, the PRR party (PVV) in the Netherlands did not make welfare an important issue. Their leader, Geert Wilders, was not a supporter of welfare protection and downplayed its salience. However, the PVV always seemed to side with the right in reducing welfare for immigrants and allied with the left when it came to maintaining welfare programs for citizens.^12^ Fleur Agema, the only female MP within the party, fought ardently to uphold the various faculties of the welfare state,^22^ in particular funding for elderly care.

In summary, it can be seen that when the PRR choose to do something concerning health or social welfare it generally amounts to restricting access and benefits to migrants. The PVV and DFP showed a tendency to welfare chauvinism, while the Italian, Swiss and Austrian PRR parties have opted for liberal chauvinism.

## Conclusion

PRR parties have been moving from the margins to the mainstream. The mainstream parties feel threatened and in turn have adapted more nativist, authoritarian and populist sentiments, most specifically with regard to migration.^23^^,^^24^ The result is a shift toward ethnocentrist politics in Europe that we should expect to have consequences for the health of citizens and noncitizens alike.

Based on the cases discussed here we can expect that a PRR party will have two kinds of effects on policy. First, in most cases they de-emphasize the issue, preferring to focus on migration, crime and security rather than health and welfare. Second, they pursue exclusionary policies, whether welfare chauvinist or exclusionary liberal. These effects arise in part because the constraints of coalition government, in all of the countries with the PRR in government, have limited the areas where the PRR can have an impact. What is not clear is whether PRR parties actually deliver better benefits for citizens. The impact of the PRR has so far been felt primarily by non-citizens such as refugees and migrants, but the benefit to indigenous citizens voting for the PRR has been limited. To a migrant, or anybody who might be mistaken for one, the PRR parties in government certainly make a difference. To those who see the PRR parties shoring up and shaping conservative governments, parties certainly make a difference.
